# Oxidative stress mediates thalidomide-induced pain by targeting peripheral TRPA1 and central TRPV4

**DOI:** 10.1186/s12915-020-00935-9

**Published:** 2020-12-14

**Authors:** Francesco De Logu, Gabriela Trevisan, Ilaria Maddalena Marone, Elisabetta Coppi, Diéssica Padilha Dalenogare, Mustafa Titiz, Matilde Marini, Lorenzo Landini, Daniel Souza Monteiro de Araujo, Simone Li Puma, Serena Materazzi, Gaetano De Siena, Pierangelo Geppetti, Romina Nassini

**Affiliations:** 1 Department of Health Sciences, Section of Clinical Pharmacology and Oncology, University of Florence, Viale Pieraccini 6, 50139 Florence, Italy; 2 Graduate Program in Pharmacology, Federal University of Santa Maria (UFSM), Santa Maria, Brazil; 3 Department of Neuroscience, Psychology, Drug Research and Child Health (NEUROFARBA), Section of Pharmacology and Toxicology, University of Florence, Viale Pieraccini 6, Florence, Italy

**Keywords:** Thalidomide, Oxidative stress, TRPA1, TRPV4, Chemotherapeutic-induced peripheral neuropathy

## Abstract

**Background:**

The mechanism underlying the pain symptoms associated with chemotherapeutic-induced peripheral neuropathy (CIPN) is poorly understood. Transient receptor potential ankyrin 1 (TRPA1), TRP vanilloid 4 (TRPV4), TRPV1, and oxidative stress have been implicated in several rodent models of CIPN-evoked allodynia. Thalidomide causes a painful CIPN in patients via an unknown mechanism. Surprisingly, the pathway responsible for such proalgesic response has not yet been investigated in animal models.

**Results:**

Here, we reveal that a single systemic administration of thalidomide and its derivatives, lenalidomide and pomalidomide, elicits prolonged (~ 35 days) mechanical and cold hypersensitivity in C57BL/6J mouse hind paw. Pharmacological antagonism or genetic deletion studies indicated that both TRPA1 and TRPV4, but not TRPV1, contribute to mechanical allodynia, whereas cold hypersensitivity was entirely due to TRPA1. Thalidomide per se did not stimulate recombinant and constitutive TRPA1 and TRPV4 channels in vitro, which, however, were activated by the oxidative stress byproduct, hydrogen peroxide. Systemic treatment with an antioxidant attenuated mechanical and cold hypersensitivity, and the increase in oxidative stress in hind paw, sciatic nerve, and lumbar spinal cord produced by thalidomide. Notably, central (intrathecal) or peripheral (intraplantar) treatments with channel antagonists or an antioxidant revealed that oxidative stress-dependent activation of peripheral TRPA1 mediates cold allodynia and part of mechanical allodynia. However, oxidative stress-induced activation of central TRPV4 mediated the residual TRPA1-resistant component of mechanical allodynia.

**Conclusions:**

Targeting of peripheral TRPA1 and central TRPV4 may be required to attenuate pain associated with CIPN elicited by thalidomide and related drugs.

## Background

Thalidomide is an old sedative, anti-emetic, and anxiolytic drug, withdrawn from the market because it causes teratogenicity [1]. Its clinical use has been repurposed for the treatment of complications of leprosy [2] and, as an alternative to bortezomib, for multiple myeloma, other hematological malignancies, and solid tumors [3]. However, like other chemotherapeutics, the anticancer action of thalidomide is associated with the development of a painful peripheral neuropathy that may result in delay or even premature termination of an otherwise successful treatment [4]. The thalidomide derivatives, lenalidomide and pomalidomide, have also been reported to cause painful neuropathy [5, 6]. Paradoxically, several studies have shown analgesic properties of thalidomide in mouse models of inflammatory [7], cancer [8], and neuropathic [9] pain. A variety of mechanisms have been proposed to explain the analgesic activity of thalidomide, including downregulation of the tumor necrosis factor-α (TNF-α) [7, 9], and inhibition of nuclear factor kappa B (NF-κB) expression [8]. A paper [10] reported electrophysiological conduction abnormalities in primary sensory neurons of thalidomide-treated rats. However, to the best of our knowledge, there are no established animal models of thalidomide-induced neuropathy which reproduce painful responses in rodents, mimicking those that they cause in patients.

The transient receptor potential ankyrin 1 (TRPA1) channel is abundantly expressed by a subpopulation of primary sensory neurons [11]. Pharmacological blockade and genetic deletion of TRPA1 completely abrogated mechanical and cold hypersensitivity induced by the proteasome inhibitor, bortezomib, and platinum-based anticancer drugs (cisplatin and oxaliplatin) in rodents [12, 13]. Among the additional TRP channels expressed in primary sensory neurons, vanilloid 4 (TRPV4) [14] has been implicated in mechanical hypersensitivity produced by paclitaxel in mice [15] and TRPV1 has been found to contribute to cisplatin-induced thermal hyperalgesia [16]. Although TRPA1 is a major oxidant sensor [17], as it is activated by an unprecedented series of reactive and electrophilic substances, including hydrogen peroxide (H_2_O_2_) and 4-hydroxynonenal (4-HNE) [18, 19], TRPV1 and TRPV4 are also sensitive to the redox potential of the milieu [17].

Ever-increasing evidence indicates that reactive oxygen species (ROS) sustain pain hypersensitivity in a variety of neuropathic pain models, including diabetic neuropathy [20], alcohol-related peripheral neuropathy [21], peripheral nerve injury [22, 23], and chemotherapeutic-induced peripheral neuropathy (CIPN) [12, 13, 15, 24, 25]. Treatment with different classes of anticancer drugs, including platinum salts, bortezomib, and spindle poisons (vinca alkaloids, taxanes, epothilones), produces oxidative stress [26]. This response contributes to their anticancer action, but seems to be responsible for significant side effects, including CIPN [27]. In line with the assumption that ROS contribute to CIPN, several preclinical findings have shown that mechanical and thermal hypersensitivity evoked in rodents by chemotherapeutics is attenuated by antioxidants [28]. However, these positive results have not been replicated by clinical studies [29, 30]. Failure of antioxidants to alleviate CIPN might be attributed to their rapidly exhausted antioxidant activity. For this reason, the identification of the possible targets that mediate the proalgesic action of ROS in CIPN is of marked interest.

The aim of the present study was twofold. First, we explored whether thalidomide, lenalidomide, and pomalidomide evoked pain-like responses in mice. Second, as thalidomide administration is known to generate oxidative stress in mice, rats, and humans [31, 32], we explored the role of oxidative stress and TRP channels sensitive to oxidants in mechanical and thermal hypersensitivities evoked by thalidomide, lenalidomide, and pomalidomide. Via pharmacological and genetic tools, we revealed that the three drugs produce mechanical and cold hypersensitivity. We also found that oxidative stress generated in peripheral tissues targets TRPA1 to signal cold allodynia, and part of the mechanical allodynia, whereas oxidative stress generated in the central nervous system (CNS) targets central TRPV4 to mediate the TRPA1-resistant component of mechanical allodynia.

## Results

### Thalidomide evokes mechanical and cold hypersensitivity mediated by TRPA1 and TRPV4

**Fig. 1:**
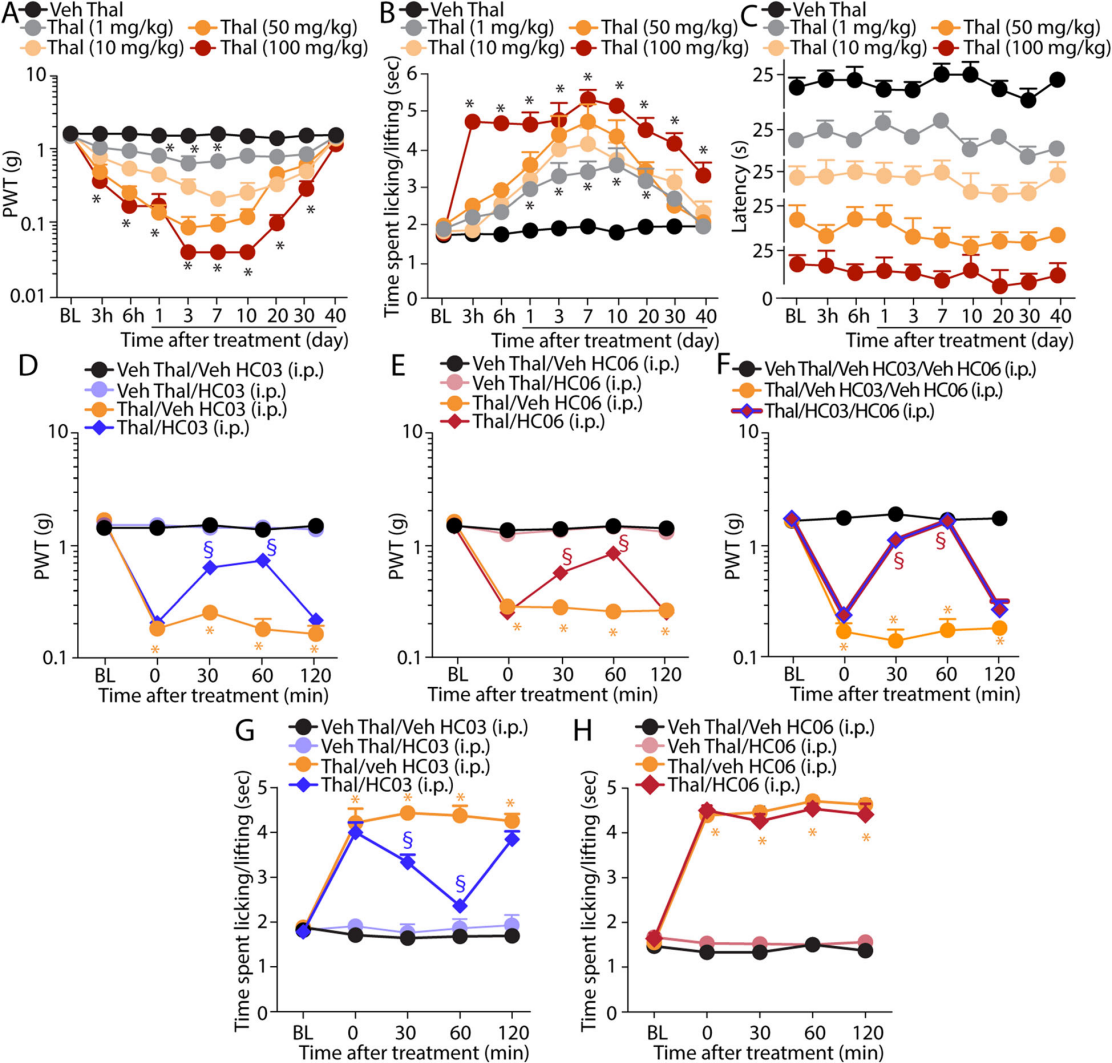
Thalidomide elicits mechanical and cold, but not heat, hypersensitivity that is dependent on TRPA1 and TRPV4. **a**–**c** Dose- and time-dependent mechanical and cold allodynia and heat hypersensitivity following intraperitoneal (i.p.) injection of thalidomide (Thal, 1, 10, 50, and 100 mg/kg) or Veh. **d**–**f** Mechanical allodynia at day 7 following Thal (50 mg/kg, i.p.) and Veh and after the administration of HC-030031 (HC03, 100 mg/kg, i.p.), HC-067047 (HC06, 10 mg/kg, i.p.), a combination of HC03 (100 mg/kg, i.p.) and HC06 (10 mg/kg, i.p.), or Veh. **g**, **h** Cold allodynia at day 7 following Thal (50 mg/kg, i.p.) or Veh and after the administration of HC03 (100 mg/kg, i.p.), HC-067047 (HC06, 10 mg/kg, i.p.), or Veh. BL, baseline. Data are mean ± SEM, *n* = 6 mice. *P < 0.05 vs. Veh Thal or Veh Thal/Veh HC03/Veh HC06; ^§^*P* < 0.05 vs. Thal/Veh HC03/Veh HC06. Two-way ANOVA followed by Bonferroni’s post hoc test

To explore whether thalidomide elicited sensory hypersensitivities in mice, we administered a single i.p. injection of increasing doses (1, 10, 50, and 100 mg/kg) of the drug, or its vehicle, in C57BL/6J mice. We observed a dose-dependent, early (3 h after administration), and prolonged (~ 35 days) mechanical and cold allodynia (Fig. 1a, b). In contrast, any dose of thalidomide failed to evoke hypersensitivity to thermal (hot) stimuli (Fig. 1c). Further mechanistic studies were performed in mice treated with a single dose (50 mg/kg, i.p.) of thalidomide, which, after the man to mouse conversion [33], approximates the starting therapeutic dose (200 mg) used in patients [34].

To test the implication of TRP channels, both pharmacological and genetic tools were used. Seven days after thalidomide administration, when the allodynia plateaued, systemic (i.p.) administration of the selective TRPA1 antagonist, HC-030031 (100 mg/kg) [35], partially reversed mechanical allodynia (Fig. 1d), without affecting the basal threshold value in naive animals (Fig. 1d). Because of the incomplete inhibition produced by TRPA1 antagonism, the role of TRPV4 and TRPV1 was explored. The TRPV1 antagonist, capsazepine (4 mg/kg, i.p.), given at day 7 after thalidomide or vehicle, failed to affect mechanical allodynia (Additional file 1: Fig. S1A). Conversely, systemic (i.p.) administration of the selective TRPV4 antagonist, HC-067047 (10 mg/kg, at day 7 after thalidomide or vehicle) [36], which did not affect the baseline threshold value in vehicle-treated animals (Fig. 1e), partially attenuated mechanical allodynia (Fig. 1e). However, a combination of TRPA1 and TRPV4 antagonists completely reversed thalidomide-evoked mechanical allodynia (Fig. 1f). Cold allodynia, induced by thalidomide, resulted to be exclusively dependent on TRPA1 as HC-030031 administered at day 7 after thalidomide completely attenuated the response to the cold stimulation, while administration of HC-067047 or capsazepine was ineffective (Fig. 1g, h and Additional file 1: Fig. S1B).

**Fig. 2:**
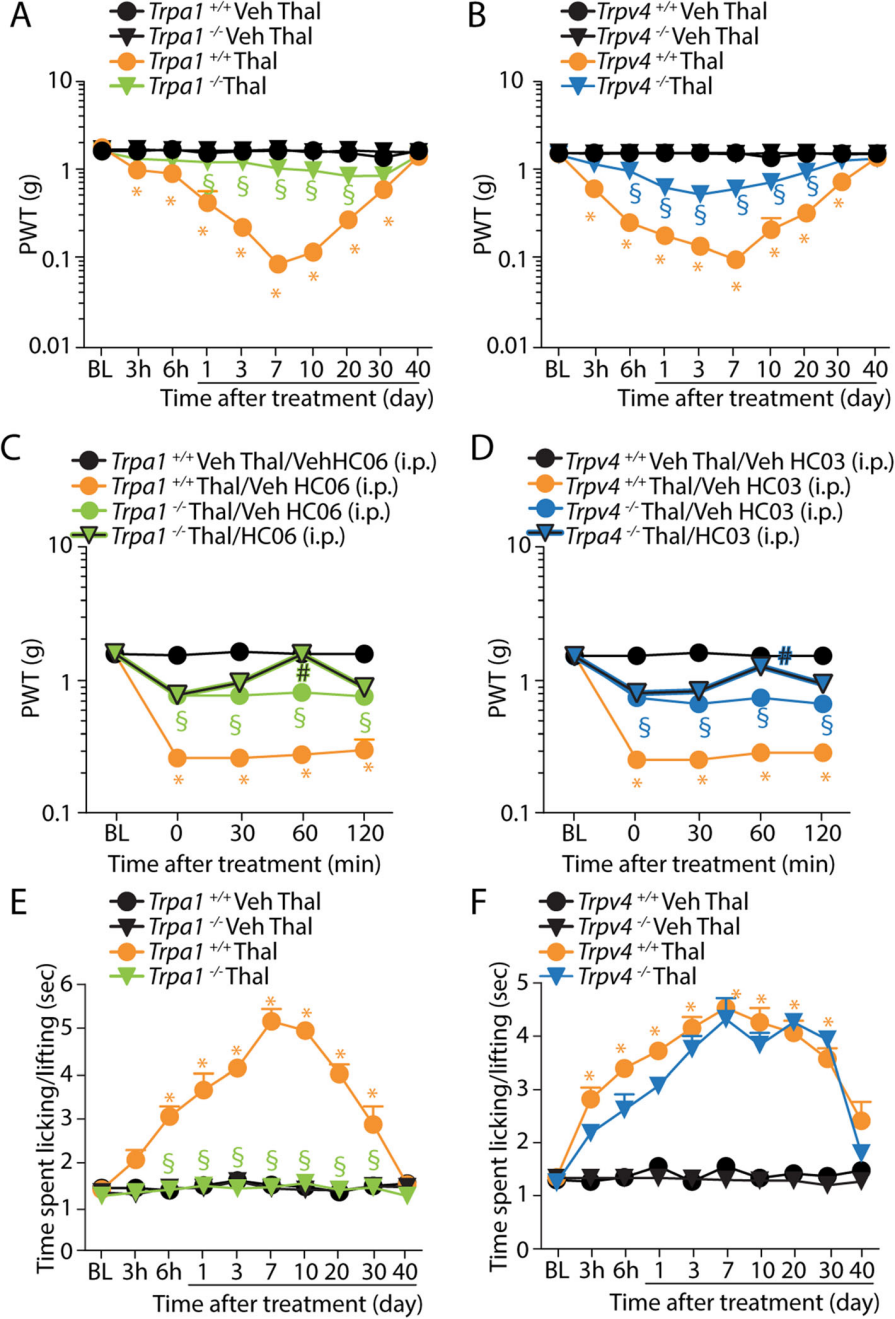
Genetic deletion of TRPA1 and TRPV4 attenuates mechanical and cold hypersensitivity evoked by thalidomide*.* **a**, **b** Time-dependent mechanical allodynia following intraperitoneal (i.p.) injection of thalidomide (Thal, 50 mg/kg) or Veh in *Trpa1*^*+/+*^, *Trpa1*^*−/−*^, *Trpv4*^*+/+*^, and *Trpv4*^*−/*−^ mice. **c**, **d** Mechanical allodynia in *Trpa1*^*+/+*^ and *Trpa1*^*−/−*^, *Trpv4*^*+/+*^ and *Trpv4*^*−/*−^ mice at day 7 following Thal (50 mg/kg, i.p.) or Veh and after the administration of HC-067047 (HC06, 10 mg/kg, i.p.) or Veh in *Trpa1*^*−/−*^ mice and HC-030031 (HC03, 100 mg/kg, i.p.) or Veh in *Trpv4*^*−/*−^ mice. **e**, **f** Time-dependent cold allodynia following Thal (50 mg/kg, i.p.) or Veh in *Trpa1*^*+/+*^, *Trpa1*^*−/−*^, *Trpv4*^*+/+*^, or *Trpv4*^*−/*−^ mice. BL, baseline. Data are mean ± SEM, *n* = 6 mice. *P < 0.05 vs. *Trpa1*^*+/+*^/Veh Thal or *Trpv4*^*+/+*^/Veh Thal or *Trpa1*^*+/+*^/Veh Thal/Veh HC06 or *Trpv4*^*+/+*^/Veh Thal/Veh HC03; ^§^*P* < 0.05 vs. *Trpa1*^*+/+*^/Thal or *Trpv4*^*+/+*^/Thal or *Trpa1*^*+/+*^/Thal/Veh HC06 or *Trpv4*^*+/+*^/Thal/Veh HC03; ^#^*P* < 0.05 vs. *Trpa1*^*−/−*^/Thal/Veh HC06 or *Trpv4*^*−/−*^/Thal/Veh HC03. Two-way ANOVA followed by Bonferroni’s post hoc test

To further prove the contribution of TRP channels, mice with genetic deletion of TRPA1, TRPV4, or TRPV1 were used. *Trpa1*^*+/+*^, *Trpv4*^*+/+*^, and *Trpv1*^*+/+*^ mice developed mechanical and cold hypersensitivity with time courses similar to those observed in C57BL/6J mice, starting 3 h and lasting ~ 35 days after thalidomide administration (Fig. 2a, b and Additional file 1: Fig. S1C). While *Trpv1*^−/−^ mice showed unchanged mechanical hypersensitivities (Additional file 1: Fig. S1C), in *Trpa1*^*−/−*^ and *Trpv4*^*−/−*^ mice, thalidomide-evoked mechanical allodynia was significantly, but not completely, reduced (Fig. 2a, b). The relative contribution of TRPA1 and TRPV4 to thalidomide-evoked mechanical allodynia was further investigated by evaluating the combined effect of channel pharmacological antagonism and genetic deletion. Thus, mechanical allodynia at day 7 after thalidomide injection was completely attenuated in *Trpa1*^−/−^ treated with HC-067047 (10 mg/kg, i.p.) and in *Trpv4*^−/−^ mice treated with HC-030031 (100 mg/kg, i.p.) (Fig. 2c, d). Cold allodynia observed in *Trpa1*^*+/+*^, *Trpv4*^*+/+*^, and *Trpv1*^*+/+*^ was completely abolished in *Trpa1*^−/−^ mice and unaffected in both *Trpv4*^−/−^ and *Trpv1*^*−/−*^ mice (Fig. 2e, f and Additional file 1: Fig. S1D). Similar results, for either mechanical or cold allodynia, were obtained if a lower dose of thalidomide (1 mg/kg) was tested in the three strains of mice (Additional file 1: Fig. S1E-G).

### Pomalidomide and lenalidomide evoke mechanical and cold allodynia similar to thalidomide

The two newer derivatives of thalidomide, pomalidomide and lenalidomide, used for the treatment of multiple myeloma and other hematological conditions [37], have been reported to evoke CIPN [5, 6]. Thus, the ability of pomalidomide and lenalidomide to induce mechanical and thermal hypersensitivity was explored in mice. Systemic administration of amounts of pomalidomide (1 mg/kg, i.p.) and lenalidomide (5 mg/kg, i.p.), equivalent in mice to the respective therapeutic doses [6, 38], induced a time-dependent mechanical and cold allodynia that initiated 3 h and lasted 35 days after drug administration (Additional file 1: Fig. S2A and B). The two drugs did not affect the threshold value to heat stimuli (Additional file 1: Fig. S2C). Thalidomide, pomalidomide, and lenalidomide did not affect motor coordination and balance in mice, which were evaluated by using the rotarod and the balance beam walk tests (Additional file 1: Fig. S2D and S2E). In addition, mice did not exhibit writhing or other stereotypic behaviors, such as freezing or hyperactivity, curling, grooming, or biting/licking, after drug injection. Seven days after pomalidomide and lenalidomide administration, treatment with HC-030031 (100 mg/kg, i.p.) or HC-067047 (10 mg/kg, i.p.) partially reversed mechanical allodynia (Additional file 1: Fig. S2F and S2G), which was, however, completely attenuated in mice receiving a combination of HC-030031 and HC-067047 (Additional file 1: Fig. S2H). Cold allodynia evoked by pomalidomide and lenalidomide was entirely inhibited by HC-030031 and a combination of HC-030031 and HC-067047, whereas HC-067047 was ineffective (Additional file 1: Fig. S2J-S2L). Present pharmacological and genetic findings indicate that TRPA1 and TRPV4 channels contribute to the mechanical allodynia induced by thalidomide and its derivatives, but only TRPA1 mediates cold hypersensitivity caused by these drugs.

### Thalidomide and its derivatives elicit hypersensitivity via oxidative stress generation that targets TRPA1 and TRPV4

**Fig. 3:**
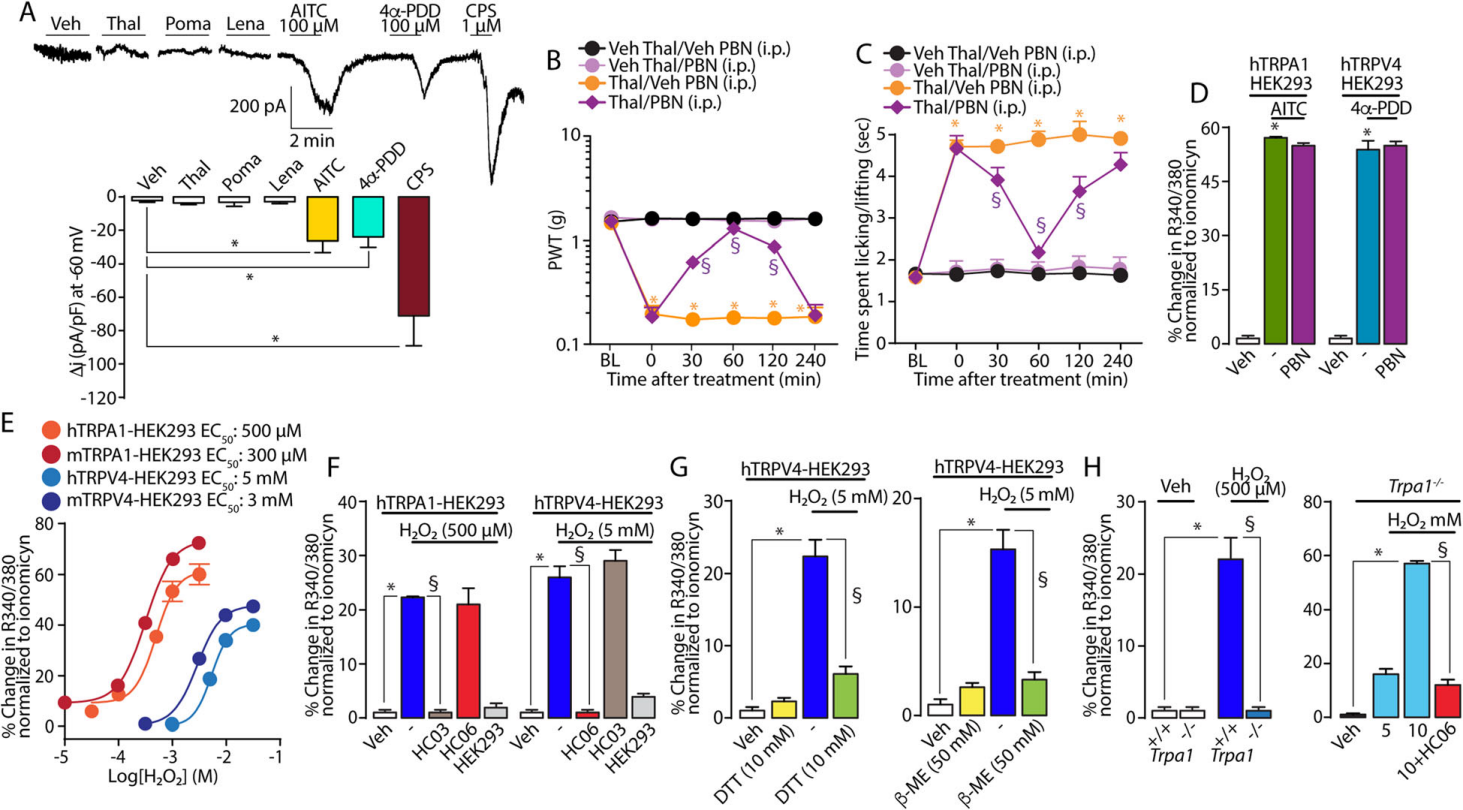
Oxidative stress targets TRPA1 and TRPV4. **a** Typical traces and pooled data of patch-clamp inward currents elicited by thalidomide (Thal), pomalidomide (Poma) and lenalidomide (Lena) (all, 100 μM), allyl isothiocyanate (AITC, 100 μM), 4α-phorbol 12,13-didecanoate (4α-PDD, 100 μM), capsaicin (CPS, 1 μM), or Veh in mouse dorsal root ganglion (DRG) neurons. Data are mean ± SEM, *n* = 4–5 cells. *P < 0.05 vs. Veh. One-way ANOVA followed by Bonferroni’s post hoc test. **b**, **c** Mechanical and cold allodynia at day 7 following the intraperitoneal (i.p.) injection of thalidomide (Thal, 50 mg/kg) or Veh, and after the administration of phenyl-α-tert-butyl nitrone (PBN, 100 mg/kg, i.p.) or Veh. Data are mean ± SEM, *n* = 6 mice. *P < 0.05 vs. Veh Thal/Veh PBN; ^§^*P* < 0.05 vs. Thal/Veh PBN. Two-way ANOVA followed by Bonferroni’s post hoc test. **d** Pooled data of Ca^2+^ response to AITC (10 μM), 4α-PDD (1 μM), or Veh hTRPA1-HEK293 and hTRPV4-HEK293 cells in the presence of PBN (30 μM). **e** Concentration response (Ca^2+^ mobilization)-curve to H_2_O_2_ in cultured hTRPA1- and mTRPA1-HEK293 and hTRPV4- and mTRPV4-HEK293 transfected cells. **f** Pooled data of Ca^2+^ response to H_2_O_2_ (500 μM and 5 mM) or Veh in untransfected HEK293 or hTRPA1-HEK293, hTRPV4-HEK293 cells in the presence of HC-030031 (HC03, 30 μM) or HC-067047 (HC06, 10 μM). **g** Pooled data of the Ca^2+^ response to H_2_O_2_ (5 mM) in hTRPV4-HEK293 cells in the presence of dithiothreitol (DTT, 10 mM) or 2-mercaptoethanol (β-ME, 50 mM). **h** Pooled data of the Ca^2+^ response to H_2_O_2_ (500 μM) in DRG neurons from *Trpa1*^*+/+*^ and *Trpa1*^*−/−*^ mice and H_2_O_2_ (5 and 10 mM) in DRG neurons from *Trpa1*^*−/−*^ mice in the presence of HC06 (10 μM). Data are mean ± SEM, *n* = 20–25 neurons or 80–100 cells. *P < 0.05 vs. Veh; ^§^*P* < 0.05 vs. H_2_O_2_ (500 μM, 5 mM, or 10 mM). One-way ANOVA followed by Bonferroni’s post hoc test

To test the hypothesis that thalidomide and its derivatives directly activate both the TRPA1 and TRPV4 receptors, we studied the ability of the drugs to elicit an inward current in cultured rat DRG neurons. Thalidomide, pomalidomide, and lenalidomide (all 100 μM) failed to evoke any inward current in capsaicin-sensitive DRG neurons (Fig. 3a), which otherwise responded to the TRPA1 and TRPV4 agonist, AITC (100 μM) and 4-αPDD (100 μM), respectively. Like other anticancer drugs, thalidomide and its derivatives are known to generate oxidative stress [39, 40]. Thus, we hypothesized that oxidative stress burst, and its reactive byproducts generated by thalidomide, pomalidomide, and lenalidomide, could be implicated in mechanical and cold allodynia evoked by the anticancer drug. Systemic (i.p.) administration of the ROS scavenger, PBN (100 mg/kg), at day 7 after the administration of the three drugs abated mechanical and cold allodynia (Fig. 3b, c and Additional file 1: Fig. S2I and S2M), thus supporting a role of oxidative stress. PBN did not affect the basal threshold value in naive animals. Furthermore, by using calcium imaging assay, we showed that PBN had no direct effect on TRPA1 and TRPV4 channel activity, since the calcium response evoked by channel-selective agonists, AITC and 4-αPDD, was unaffected after preincubation with PBN in hTRPA1- and hTRPV4-HEK293 cells (Fig. 3d).

While strong evidence has been accumulated on the ability of H_2_O_2_ to activate TRPA1 [18], only H_2_O_2_-mediated activation of TRPV4 was reported in human and rodent (rat and mouse) lung microvascular endothelial cells [41] and indirectly in rat brain slices [42]. Here, we show that H_2_O_2_ elicited concentration-dependent Ca^2+^ responses either in hTRPV4- or in mTRPV4-HEK293 cells with an EC_50_ of 5 mM and 3 mM, respectively, which resulted 10 times higher than that observed in hTRPA1- and mTRPA1-HEK293 cells (EC_50_ 500 μM and 300 μM, respectively) (Fig. 3e). The Ca^2+^ response evoked by a lower H_2_O_2_ concentration (500 μM) in hTRPA1-HEK293 was inhibited in the presence of HC-030031, but not of HC-067047 (Fig. 3f). However, the Ca^2+^ response evoked by H_2_O_2_ (5 mM) in hTRPV4-HEK293 was attenuated by HC-067047, but not by HC-030031. H_2_O_2_ (500 μM or 5 mM) was ineffective in untransfected HEK293 cells (Fig. 3f). Considering that H_2_O_2_ caused TRPA1 activation via the oxidization of cysteine residues [18], we tested the hypothesis that oxidation by H_2_O_2_ of TRPV4 implicates cysteine residues. In hTRPV4-HEK293, exposure to two cysteine-reducing agents, DTT and β-ME [18], attenuated the Ca^2+^ response evoked by H_2_O_2_ in hTRPV4-HEK293 cells (Fig. 3g). Finally, we tested low (500 μM) and high (5 and 10 mM) H_2_O_2_ concentrations in cultured mouse DRG neurons taken from *Trpa1*^*+/+*^ and *Trpa1*^*−/−*^ mice. The lower H_2_O_2_ concentration (500 μM) elicited a Ca^2+^ response in neurons from *Trpa1*^*+/+*^ mice, but not in those from *Trpa1*^*−/−*^ mice (Fig. 3h). The residual calcium response to a higher concentration of H_2_O_2_ (10 mM) observed in DRG neurons from *Trpa1*^*−/−*^ mice was abated in the presence of HC-067047 (Fig. 3h). Thus, in vitro data confirmed the ability of H_2_O_2_ to target the TRPV4 channel, provided that the concentration/dose of H_2_O_2_ is sufficiently high.

### Peripheral and central (spinal) TRPA1 and TRPV4 activation differentially contributes to thalidomide-induced mechanical allodynia

**Fig. 4:**
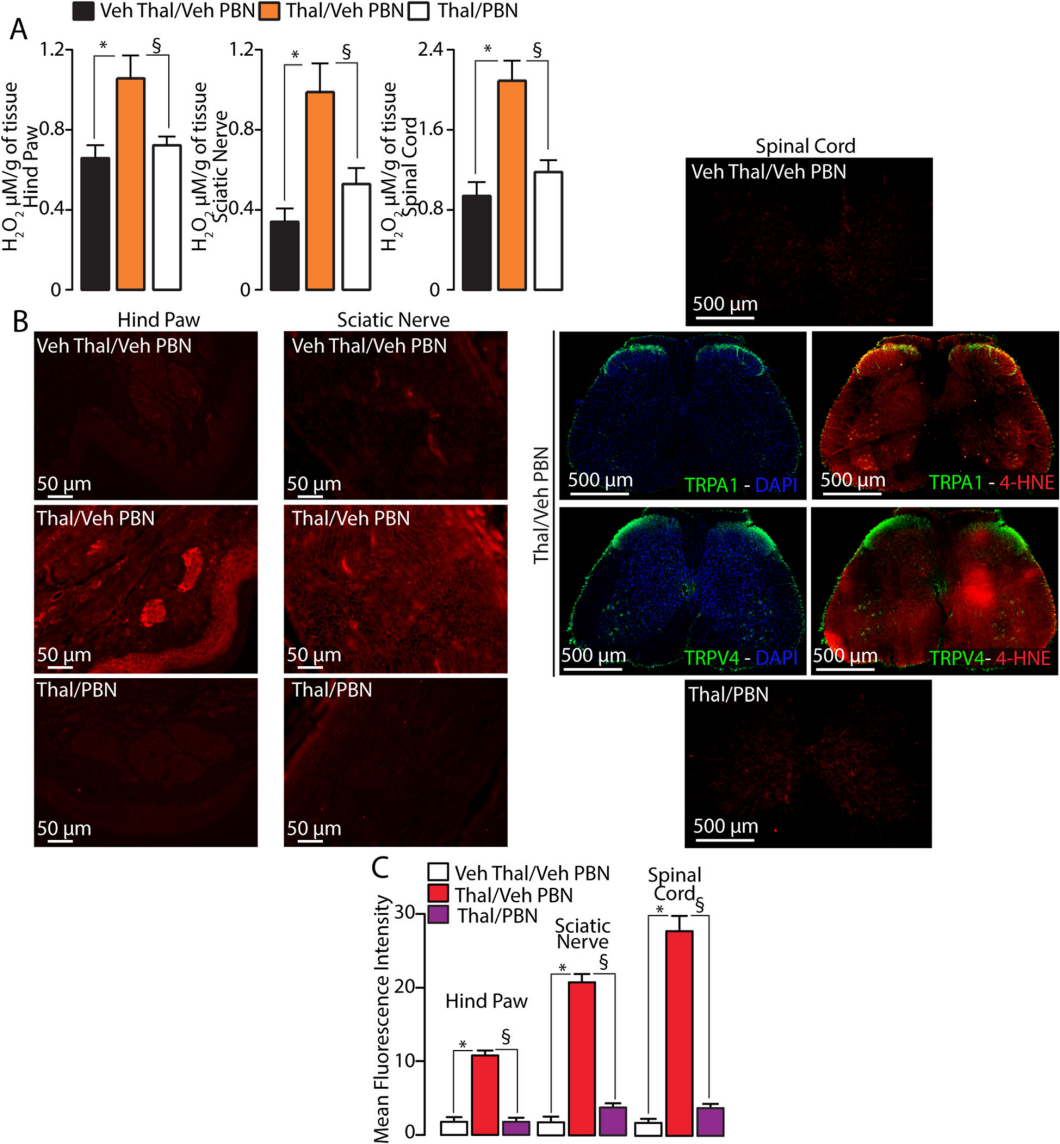
Thalidomide increases oxidative stress in the hind paw, sciatic nerve, and spinal cord. **a** H_2_O_2_ levels in the hind paw, sciatic nerve, and lumbar (L4–L6) spinal cord at day 7 following intraperitoneal (i.p.) injection of thalidomide (Thal, 50 mg/kg) or Veh and 60 min after the administration of phenyl-α-tert-butyl nitrone (PBN, 100 mg/kg, i.p.) or Veh. **b**, **c** Representative images and mean fluorescence intensity of 4-hydroxynonenal (4-HNE) staining in the hind paw, sciatic nerve, and lumbar (L4–L6) spinal cord and TRPA1 and TRPV4 staining in the spinal cord, at day 7 following Thal (50 mg/kg, i.p.) and 60 min after the administration of PBN (100 mg/kg, i.p.) or Veh. Data are mean ± SEM (*n* = 6 mice). *P < 0.05 vs. Veh Thal/Veh PBN; ^§^*P* < 0.05 vs. Thal/Veh PBN*.* One-way ANOVA followed by Bonferroni’s post hoc test

One major issue raised by the present data is that, while oxidative stress inhibition completely attenuated mechanical allodynia, TRPA1 or TRPV4 pharmacological antagonism or gene deletion provided partial reduction, and total reduction was attained solely by the simultaneous inhibition of both channels. A recent study reported that oxidative stress generated at central or peripheral sites may contribute differently to cisplatin- and paclitaxel-evoked hypersensitivity [25]. Thus, we hypothesized whether oxidative stress activates TRPA1 and TRPV4 at different anatomical sites to mediate thalidomide-evoked mechanical allodynia. To test this hypothesis, we measured two oxidative stress biomarkers, H_2_O_2_ and the more stable peroxidation product of plasma membrane phospholipid peroxidation, 4-HNE [19]. H_2_O_2_ levels (Fig. 4a) and 4-HNE staining (Fig. 4b, c) were increased in homogenates or tissue slices, respectively, of the hind paw, sciatic nerve, and lumbar spinal cord, taken from mice at day 7 after thalidomide, compared to its vehicle. Systemic treatment with a dose of PBN that reversed thalidomide-evoked allodynia reduced H_2_O_2_ levels and 4-HNE staining in all three tissues (Fig. 4a–c). Notably, 4-HNE staining in the spinal cord revealed that the oxidative stress marker does not localize to a specific site (e.g., superficial lamina) where TRPA1 and TRPV4 are mainly expressed. The 4-HNE staining was evenly distributed in the entire tissue slice of the lumbar spinal cord, thus suggesting the possibility that thalidomide generates oxidative stress in a non-specific manner from several cell types. Thus, while oxidative stress produced at each site may potentially contribute to thalidomide-evoked mechanical allodynia, the role of centrally vs. peripherally generated oxidative stress is unknown.

**Fig. 5:**
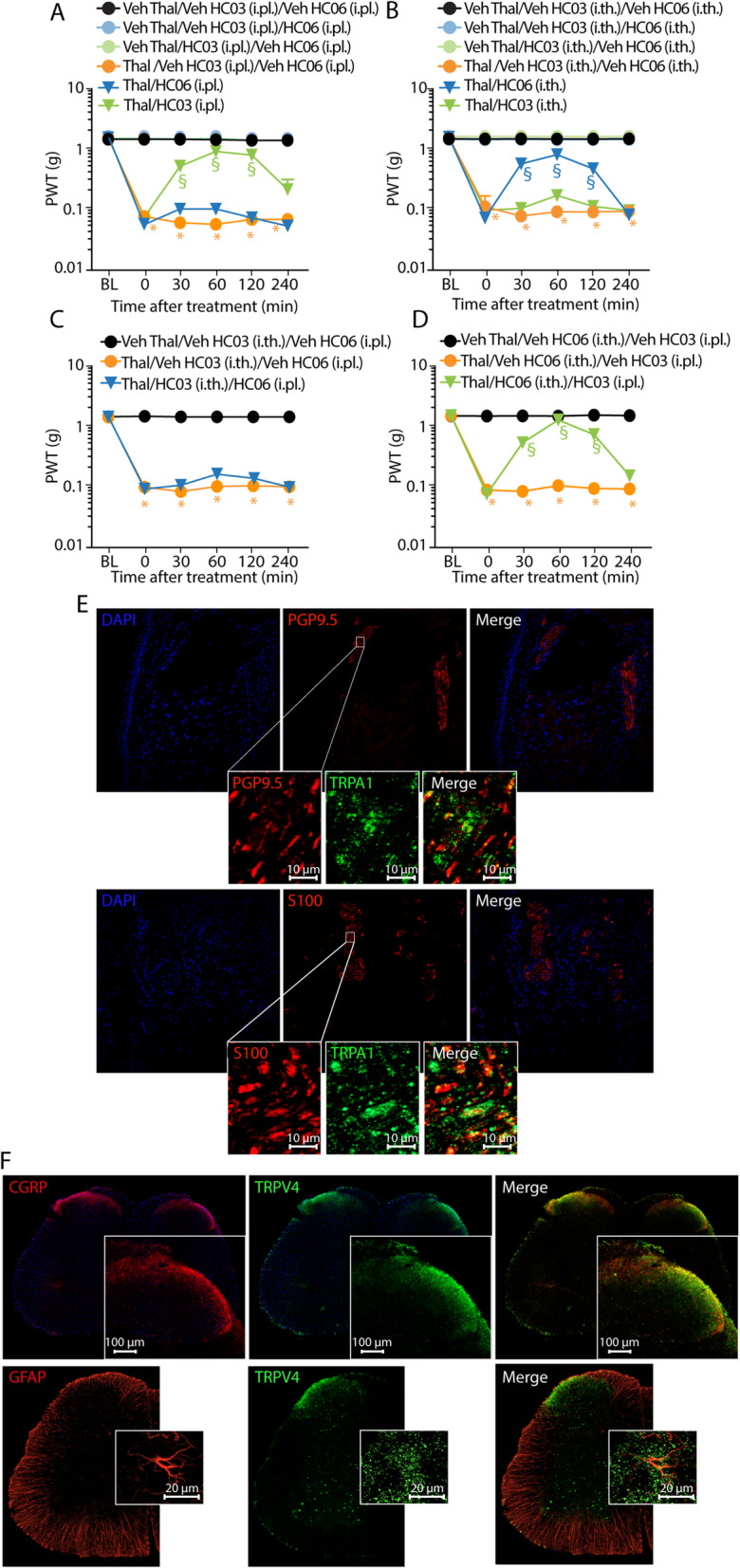
Peripheral TRPA1 and central TRPV4 contribute to thalidomide-induced mechanical allodynia. **a**, **b** Mechanical allodynia at day 7 following intraperitoneal (i.p.) thalidomide (Thal, 50 mg/kg) or Veh and after the administration of intraplantar (i.pl., 20 μl) or intrathecal (i.th., 5 μl) HC-030031 (HC03, 100 μg), HC-067047 (HC06, 100 μg), or Veh. **c**, **d** Mechanical allodynia at day 7 following Thal (50 mg/kg, i.p.) or Veh and after the administration of a combination of HC03 (100 μg, i.th.) and HC06 (100 μg, i.pl.) or HC06 (100 μg, i.th.) and HC03 (100 μg, i.pl.) or Veh. **e** Representative images of co-expression of PGP9.5 or S100 and TRPA1 in the peripheral nerve of hind paw. **f** Representative images of co-expression of CGRP or GFAP and TRPV4 in the lumbar (L4–L6) spinal cord. Data are mean ± SEM, *n* = 6 mice. *P < 0.05 vs. Veh Thal/Veh HC03/Veh HC06; ^§^*P* < 0.05 vs. Thal/Veh HC03/Veh HC06. Two-way ANOVA followed by Bonferroni’s post hoc test

To explore this hypothesis, we investigated the implication of peripheral vs. central TRPA1 and TRPV4 in thalidomide-induced mechanical allodynia, by injecting channel antagonists locally in the hind paw or intrathecally in the spinal cord. We found that, at day 7 after thalidomide, mechanical allodynia was partially reversed by the i.pl. injection of HC-030031 (100 μg), but not of HC-067047 (100 μg) (Fig. 5a). In contrast, i.th. HC-067047 (100 μg), but not HC-030031 (100 μg), partially reversed thalidomide-induced mechanical allodynia (Fig. 5b). Neither i.th. nor i.pl. HC-030031 and HC-067047 affect the basal threshold value in naive animals. These results implicated the engagement of peripheral TRPA1 and central TRPV4 in the thalidomide-induced mechanical allodynia. To further support this hypothesis, at day 7 after thalidomide injection, we tested the ability of a combination of i.pl. HC-067047 and i.th. HC-030031, or vice versa, to attenuate thalidomide-evoked mechanical allodynia. While a combination of i.pl. HC-067047 and i.th. HC-030031 failed to affect allodynia (Fig. 5c), a combination of i.pl. HC-030031 and i.th. HC-067047 provided complete reversal of the pain-like response (Fig. 5d), thus supporting the view that TRPA1 mediates the peripheral, and TRPV4 the central, component of thalidomide-evoked mechanical allodynia. To better address which cell types expressing TRPA1 in the periphery and TRPV4 at the central level are involved in thalidomide-evoked pain responses, a colocalization study with double immunofluorescence staining was undertaken. In slices that contain bundles of the plantar nerve, TRPA1 expression was detected in PGP9.5^+^ (protein gene product) nerve fibers and S100^+^ Schwann cells (Fig. 5e). At the central level, TRPV4 staining exhibited a well-matched colocalization with CGRP^+^ (calcitonin gene-related peptide) nerve fibers and GFAP^+^ (glial fibrillary acidic protein) astrocytes (Fig. 5f).

**Fig. 6:**
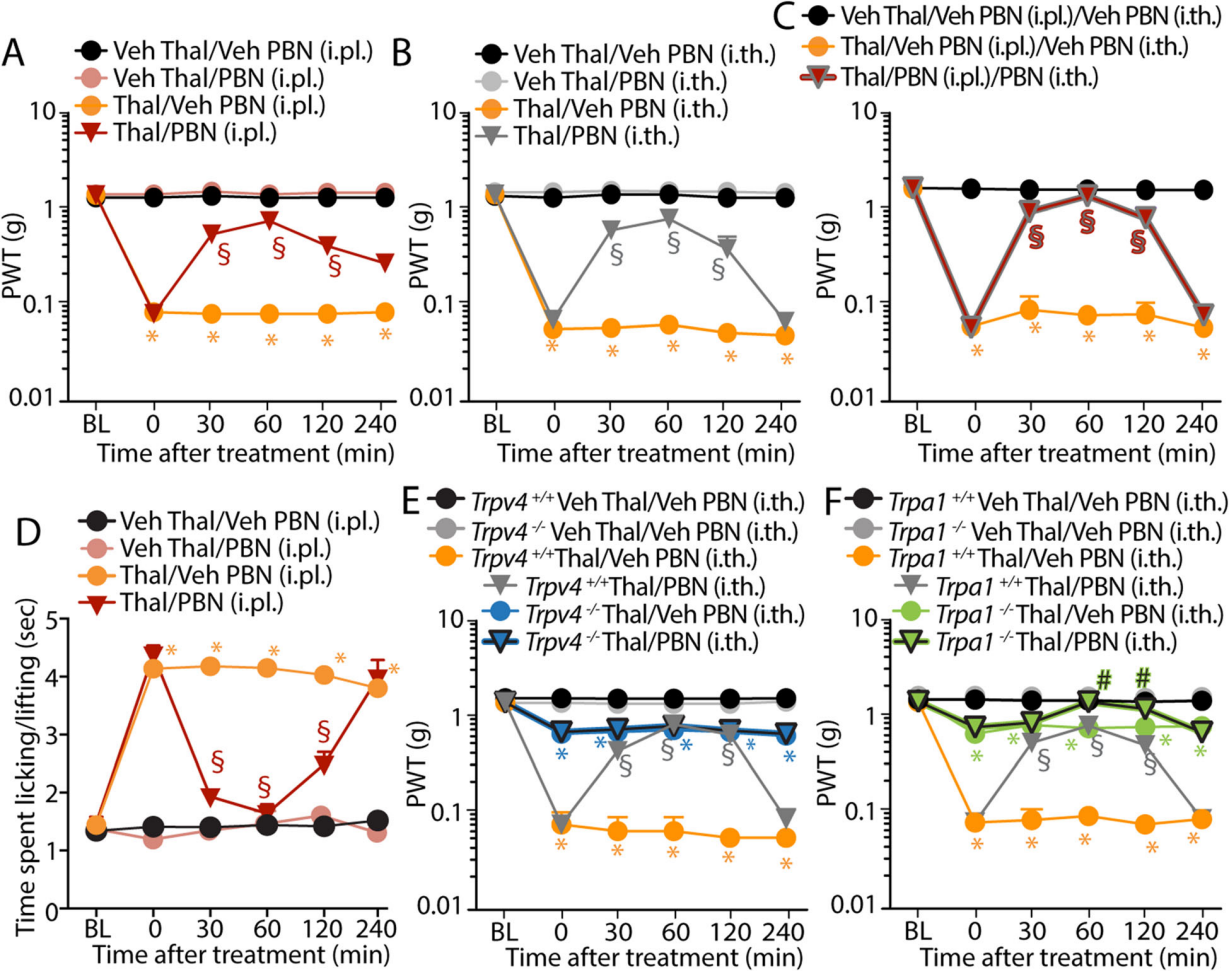
Oxidative stress targeting peripheral TRPA1 and central TRPV4 mediates thalidomide-induced mechanical allodynia. **a**–**c** Mechanical allodynia at day 7 following thalidomide (Thal, 50 mg/kg) or Veh and after the administration of i.pl. or a combination of i.pl. and i.th. phenyl-α-tert-butyl nitrone (PBN, 100 μg) or Veh. **d** Cold allodynia at day 7 following Thal (50 mg/kg, i.p.) or Veh and after the administration of i.pl. PBN (100 μg) or Veh. **e**, **f** Mechanical allodynia in *Trpv4*^*+/+*^, *Trpv4*^*−/*−^, *Trpa1*^*+/+*^, and *Trpa1*^*−/−*^ mice at day 7 following Thal (50 mg/kg, i.p.) or Veh and after the administration of i.th. PBN (100 μg) or Veh. Data are mean ± SEM, *n* = 6 mice. *P < 0.05 vs. Veh Thal/Veh PBN; ^§^*P* < 0.05 vs. Thal/Veh PBN; ^#^*P* < 0.05 vs. *Trpa1*^*−/−*^/Thal/Veh PBN. Two-way ANOVA followed by Bonferroni’s post hoc test

To understand how the increased oxidative stress could engage the peripheral TRPA1 and the central TRPV4, PBN was given to mice by either i.pl. or i.th. administration. At day 7 after thalidomide, i.pl. (100 μg) or i.th. (100 μg) PBN injection partially inhibited mechanical allodynia (Fig. 6a, b), while a combination of i.pl. and i.th. PBN completely attenuated the response (Fig. 6c). However, cold allodynia, which is entirely TRPA1-dependent, was completely reversed by i.pl. PBN (Fig. 6d). PBN (i.pl. or i.th.) did not affect the basal threshold value in vehicle-treated mice. Similar results were obtained with the H_2_O_2_-detoxifying enzyme, catalase. Either i.pl. or i.th. catalase administration partially inhibited mechanical allodynia, which, however, resulted completely reduced by a combination of i.pl. and i.th. catalase (Additional file 1: Fig. S3A-S3C). As for PBN, catalase (i.pl. but not i.th.) completely reversed cold allodynia (Additional file 1: Fig. S3D and S3E). Due to the selective activity of catalase against H_2_O_2_, these data strengthen the prominent role of H_2_O_2_ at both central and peripheral levels in thalidomide-evoked mechanical cold allodynia. We also found that i.th. PBN, while partially reversing mechanical allodynia in *Trpa1*^*+/+*^ and *Trpv4*^*+/+*^ mice (Fig. 6e, f), did not affect the residual mechanical allodynia in *Trpv4*^−/−^ (Fig. 6e), and completely reversed the residual response in *Trpa1*^−/−^ mice (Fig. 6f). Altogether, these data indicate that selective ROS scavenging at either the peripheral or the central levels inhibits the correspondent TRPA1 and TRPV4 component, respectively. Only simultaneous inhibition of the oxidative stress that targets both ion channels warrants complete attenuation of the mechanical allodynia.

## Discussion

Thalidomide, an old sedative and anti-emetic drug banned for causing birth defects in humans, has been repurposed for the treatment of leprosy and several types of cancer [2], including multiple myeloma, myelodysplastic syndrome, and several solid cancers [43]. Thalidomide derivatives, pomalidomide and lenalidomide, also exhibit anticancer activity in multiple myeloma patients who relapse or are refractory to other anticancer treatments. Unfortunately, as with other chemically unrelated chemotherapeutic agents (platinum-based drugs, taxanes, and bortezomib), thalidomide and its derivatives cause a painful peripheral polyneuropathy that often results in severe discomfort or even drug discontinuation [4]. Despite its clinical relevance, the underlying mechanism of the neuropathy and the associated pain symptoms caused by thalidomide and its derivatives remains poorly known.

Several studies have investigated the ability of thalidomide to attenuate inflammatory and neuropathic pain in rodent models presumably by interacting with indirect mechanisms dependent on the inhibition of proalgesic cytokines, such as TNF-α and NF-κB [7–9]. Surprisingly, to the best of our knowledge, no study has investigated the ability of thalidomide or related drugs to elicit pain-like responses in animal models so far. Only one study has shown that thalidomide increased electrophysiological responses in rat sensory nerve fibers [10], reminiscent of a sensory neuropathy. Thus, our study shows for the first time that thalidomide, pomalidomide, and lenalidomide evoke mechanical and cold hypersensitivity in mice. Although the chemotherapeutic drugs failed to evoke heat hypersensitivity, as cold and mechanical allodynia are the major and most debilitating symptoms of thalidomide-evoked CIPN [44, 45], the present model seems to satisfactorily replicate the human pain condition. As previously reported for other chemotherapeutic agents, including oxaliplatin, vincristine, bortezomib, and paclitaxel [12, 13, 15, 28, 46–48], we report that a single administration of thalidomide, lenalidomide, or pomalidomide is sufficient to produce a sustained condition of sensory hypersensitivity in mice, which somehow mimics the prolonged duration of CIPN in patients [5, 6]. However, the molecular and cellular mechanisms responsible for the prolonged thalidomide-evoked hypersensitive phenotype remain unknown and deserve further investigation. A series of previous investigations have highlighted the ability of thalidomide to produce beneficial and toxic effects, including its anticancer action and severe teratogenic effects, via the generation of oxidative stress [39, 40]. In particular, it has been reported that the bioactivation of thalidomide from horseradish peroxidase to free-radical intermediates produces ROS, which cause oxidative damage to DNA and other cellular macromolecules, apparently responsible for the anticancer effect, but also for the teratogenic action [40].

Our data show that oxidative stress byproducts, such as H_2_O_2_ and 4-HNE, are generated both at the peripheral (hind paw and sciatic nerve) and central (spinal cord) levels, after thalidomide systemic injection. Thus, thalidomide generates oxidative stress along the entire pain pathway, which encompasses the entire anatomical route that conveys the pain signal from the hind paw to the lumbar spinal cord. The observation that increased H_2_O_2_ and 4-HNE levels were reduced by systemic treatment with the antioxidant, PBN, further supports the hypothesis that oxidative stress is essential for the pain-like symptoms evoked by thalidomide. We also revealed that TRPA1 and TRPV4 channels mediate mechanical and cold allodynia evoked by thalidomide and related drugs. However, as reported for other anticancer drugs, including oxaliplatin/cisplatin, paclitaxel, and bortezomib [12, 13, 15], the observation that thalidomide and its derivatives failed to evoke any excitatory effect in cultured TRPA1- and TRPV4-expressing neurons excludes the possibility that their proalgesic effect depends on a direct action on these channels, and suggests the implication of indirect mechanisms, including oxidative stress generation.

Whereas the role of TRPA1 as a sensor of oxidative stress has been extensively investigated and recognized, a similar function of TRPV4 has been poorly explored. Several studies have reported that TRPA1 is activated by an unprecedented series of reactive oxygen, nitrogen, or carbonyl species [18, 19]. In particular, robust proof supports the hypothesis that H_2_O_2_ causes nociceptor stimulation via TRPA1 [22, 23]. In contrast, little evidence showing that H_2_O_2_ stimulates TRPV4 has been provided [41, 42]. TRPV4 carries cysteine residues, corresponding to those found in other redox-sensitive TRP channels [17], thus enabling their targeting through oxidation, which leads to channel activation [17]. Here, we report that H_2_O_2_ targets the recombinant and native TRPV4, although with a potency about 10 times lower than that exhibited toward TRPA1. The observation that two cysteine-reducing agents, DTT and β-ME [18, 49], abated the H_2_O_2_ evoked Ca^2+^ response further supports the hypothesis that TRPV4 may be directly activated by oxidants. The more elevated H_2_O_2_ concentrations after thalidomide in the spinal cord and the efficacy of i.th. administration of the specific H_2_O_2_ scavenger, catalase, strengthen the role of H_2_O_2_ to mediate the central TRPV4-dependent component of thalidomide-evoked mechanical allodynia.

Results obtained in cultured DRG neurons strengthen the findings obtained in recombinant systems. While the Ca^2+^ response produced by a low H_2_O_2_ concentration was entirely abated in the presence of the TRPA1 antagonist, the response to a higher H_2_O_2_ concentration was blocked only when a TRPV4 antagonist was added. Importantly, the residual Ca^2+^ response to a high H_2_O_2_ concentration observed in DRG neurons from *Trpa1*^*−/−*^ mice was completely attenuated by a TRPV4 antagonist. Thus, it may be concluded that, provided a sufficiently elevated burden is present, oxidative stress may engage not only TRPA1 but also TRPV4.

A peculiar difference of the two channels regarding their roles in thalidomide-evoked hypersensitivities is that, as shown by genetic or pharmacological studies, mechanical allodynia was partially attenuated by these interventions, and abolition was attained only by a combination of peripheral TRPA1 and central TRPV4 blockade. Various anticancer drugs, including platinum-derived drugs or the proteasome inhibitor, bortezomib, promote mechanical allodynia exclusively via oxidative stress and the ensuing TRPA1 activation [12, 13]. The partial contribution of TRPV4 to mechanical hypersensitivity has been previously reported in the CIPN model produced by the taxane derivative, paclitaxel [15]. Notably, as shown in the paclitaxel-evoked model [15], cold allodynia elicited by thalidomide, lenalidomide, and pomalidomide was entirely TRPA1-dependent. However, the reason why cold hypersensitivity is solely dependent on TRPA1, whereas mechanical allodynia requires the contribution of both TRPA1 and TRPV4, remains unknown.

We previously found that local (intraplantar) administration of an oxidative stress scavenger or a TRPA1 antagonist completely reversed mechanical and cold allodynia evoked by bortezomib and oxaliplatin [12], suggesting that TRPA1 sensitization/activation may occur in terminal nerve fibers of the hind paw. To understand more precisely where TRPV4 and TRPA1 act to mediate thalidomide-evoked mechanical and cold allodynia, site-specific strategies of drug administration were used. Results show that peripheral (intraplantar) antagonism of TRPA1 in the mouse paw provided complete reversal of cold allodynia, but only partial attenuation of mechanical allodynia. If central (intrathecal) antagonism of TRPV4 was added to the peripheral TRPA1 blockade, thalidomide-evoked mechanical allodynia was completely inhibited.

Evidence of a differential contribution of peripheral vs. central oxidative stress has been reported in cisplatin- and paclitaxel-evoked mechanical hypersensitivity [25]. Oxidative stress manipulation experiments strengthen the conclusion deriving from channel pharmacological antagonism. In fact, whereas local oxidative stress inhibition either in the hind paw or in the spinal cord provided only partial attenuation, a combination of central and peripheral oxidative stress blockade completely reversed allodynia. Final proof that mechanical allodynia was mediated by oxidative stress activation of both peripheral TRPA1 and central TRPV4 was derived from experiments with genetic channel deletion. Elimination of oxidative stress by an intrathecal antioxidant, while not further inhibiting thalidomide-evoked mechanical allodynia in *Trpv4*^−/−^ mice, completely reversed the residual response observed in *Trpa1*^−/−^ mice. These data further confirmed that full protection from thalidomide-induced mechanical allodynia can be attained by attenuating oxidative stress at both peripheral and central sites of action. Some studies have reported that early treatment with ROS scavengers or mitochondrial activity inhibitors provided a complete and sustained prevention of mechanical hypersensitivity induced by chemotherapeutic agents [12, 50–52].

These findings suggest the existence of a time soon after the exposure to chemotherapeutics that is critical in order to initiate and maintain the generation of the proalgesic oxidative stress. In clinical settings, inhibition by ROS scavengers at such an initial event may be challenging, and therefore, the attenuation of the activity of ROS targets (TRPA1 and TRPV4 channels) at the peripheral or central level could be a better therapeutic strategy. However, it should be considered that TRPA1 and TRPV4 are expressed by a series of immune and inflammatory cells [53]. In particular, elevated expression of TRPV4 has been detected in human leukocytes [54], where it regulates key functions in response to pro-inflammatory stimuli, including ROS production and cell adhesion or migration [55], and in macrophages, where it exerts a double-edged function. A pro-inflammatory function includes phagocytosis and ROS production, and an anti-inflammatory function includes secretion of pro-resolution cytokines [56]. Furthermore, under inflammatory circumstances, T cell TRPV4 facilitates the release of interferon-γ, which represents an important mediator of tumor immune escape [57]. Constitutive expression of TRPA1 mRNA and protein has been identified in mouse and human primary CD4^+^ T cells [58] and macrophages [59]. Thus, at the present stage, it is not possible to exclude that TRPV4 and/or TRPA1 inhibition may negatively affect cancer progression.

Although we have identified the role of oxidative stress, peripheral TRPA1, and central TRPV4 in mechanical and cold hypersensitivity elicited by thalidomide and related drugs in mice, several questions remain to be investigated. These include the cell types that express peripheral TRPA1 and central TRPV4 that, engaged by oxidative stress, signal allodynia. TRPA1 is known to be expressed in nociceptors [11] and in Schwann cells that surround the fibers of these neurons [22]. Here, we confirm that in mouse peripheral tissues, prominent TRPA1 protein expression is present within the nerve fibers and Schwann cells that wrap these fibers. In the CNS, TRPV4 may be present in central terminals of nociceptors [14] and astrocytes [60]. In the lumbar spinal cord, we confirm the presence of TRPV4 in CGRP-immunoreactive fibers and in astrocytes. While TRPA1 and TRPV4 expressed by nerve fibers may directly contribute to signal pain, Schwann cell TRPA1, which have been implicated in pain sensitization [22], and astrocyte TRPV4 may indirectly sustain thalidomide-evoked allodynia. However, further studies are required to identify the intracellular and molecular mechanisms implicated in the central TRPV4-dependent and peripheral TRPA1-dependent components of thalidomide-evoked mechanical allodynia in neurons and/or glial cells. Although H_2_O_2_ levels and 4-HNE staining were higher in the spinal cord than in the paw, it is not clear if these differences may explain the differential ability of oxidative stress to target TRPA1 in the peripheral tissues and TRPV4 at the central level.

## Conclusions

From a therapeutic point of view, the present results indicate the need for peripheral acting TRPA1 antagonists and blood-brain barrier-penetrating TRPV4 antagonists to treat the pain symptoms associated to CIPN evoked by thalidomide and related drugs. However, due to the pleiotropic activity of TRPA1 and TRPV4, the safety profile of channel antagonism should be carefully scrutinized, particularly regarding the impact of this therapeutic strategy on cancer outcome and on the efficacy of cancer treatment.

## Methods

### Animals

Sprague-Dawley rats (male, 75–100 g, 4–5 weeks), C57BL/6J mice (male, 20–25 g, 5 weeks) (Harlan Laboratories), wild-type (*Trpa1*^*+/+*^) and TRPA1-deficient (*Trpa1*^*−/*−^; B6129P-Trpa1^tm1Kykw/J^; Jackson Laboratories) mice (25–30 g, 6–8 weeks) [61], wild-type (*Trpv4*^*+/+*^) and TRPV4-deficient (*Trpv4*^*−/−*^) mice (25–30 g, 5–8 weeks) [62], and wild-type (*Trpv1*^*+/+*^) and TRPV1-deficient (*Trpv1*^*−/−*^; B6129X1-Trpv1^tm1Jul/J^, Jackson Laboratories) mice (25–30 g, 5–8 weeks) generated by heterozygous mice on a C57BL/6J background were used.

### Study design

Group size of *n* = 6 animals for behavioral experiments was determined by sample size estimation using G*Power (v3.1) [63] to detect size effect in a post hoc test with type 1 and 2 error rates of 5 and 20%, respectively. Allocation concealment of mice to vehicle(s) or treatment(s) group was performed using a randomization procedure http://www.randomizer.org/. Mice were housed in a temperature- and humidity-controlled vivarium (12 h dark/light cycle, free access to food and water). Behavioral experiments were done in a quiet, temperature-controlled (20–22 °C) room between 9 am and 5 pm and were performed by an operator blinded to genotype and drug treatment. Animals were anesthetized with a mixture of ketamine and xylazine (90 mg/kg and 3 mg/kg, respectively, intraperitoneal, i.p.) and euthanized with inhaled CO_2_ plus 10–50% O_2_.

C57BL/6J, *Trpa1*^*+/+*^ or *Trpa1*^*−/−*^, *Trpv4*^*+/+*^ or *Trpv4*^*−/−*^, and *Trpv1*^*+/+*^ or *Trpv1*^*−/−*^ mice were treated with thalidomide (1, 10, 50, and 100 mg/kg, i.p.), pomalidomide (1 mg/kg, i.p.), and lenalidomide (5 mg/kg, i.p.) or their vehicle. No weight loss was observed in mice after the treatment throughout the duration of the experiments. The mechanical and thermal (hot and cold) allodynia of thalidomide, pomalidomide, and lenalidomide were monitored for 40 days starting 3 h after drug administration.

Systemic (i.p.) HC-030031 (100 mg/kg), HC-067047 (10 mg/kg), phenyl-α-tert-butyl nitrone (PBN, 100 mg/kg), and capsazepine (4 mg/kg) and local (intraplantar, i.pl., 20 μl/site) and intrathecal (i.th., 5 μl/site) HC-030031 (100 μg), HC-067047 (100 μg), PBN (100 μg), and catalase (300 UI) were administered at day 7 after thalidomide, pomalidomide, or lenalidomide injection. The vehicle for catalase was 0.9% NaCl; for other drugs, the vehicle was 4% dimethyl sulfoxide, DMSO, and 4% Tween 80 in 0.9% NaCl.

### Reagents

If not otherwise indicated, all reagents were from Sigma-Aldrich. HC-030031 (2-(1,3-dimethyl-2,6-dioxo-1,2,3,6-tetrahydro-7H-purin-7-yl)-*N*-(4-isopropylphenyl) acetamide) was provided by Prof. Delia Preti (University of Ferrara, Italy). HC-067047 was from Tocris Bioscience.

### Behavioral studies

#### Rotarod test

The locomotor function, balance, and sedation of mice were assessed after drug administration. The animals were trained on a rotarod apparatus (Ugo Basile) 24 h before the test. The day of the experiment, each mouse was individually placed on the apparatus, which accelerated from 4 to 40 rpm over the trial time of 300 s. Latency to fall was evaluated and recorded for three trials.

#### Balance beam test

Fine motor coordination and balance of mice were assessed using the balance beam test as previously described [64]. Briefly, a 1-cm dowel beam was attached to two support columns 44 cm above a padded surface. At either end of the 50-cm-long beam, a 9 × 15 cm escape platform was placed. Mice were placed on the center of the beam and released. The time the mice remained on the beam was recorded, and the resulting latency to fall of three trials was averaged.

#### von Frey test

Mechanical was measured by using a series of flexible nylon von Frey calibrated filaments of increasing stiffness and the up-and-down paradigm [65]. The mechanical paw withdrawal threshold (PWT) was determined before (basal level) and after drug administration, and the response was then calculated as previously described [66].

#### Acetone test

Cold allodynia was assessed as previously described [12]. Briefly, a droplet (50 μl) of acetone was gently applied to the plantar surface of the mouse hind paw, and the time spent in elevation and licking of the plantar region was recorded over a 60-s period. Acetone was applied three times at a 10–15-min interval, and the average elevation/licking time was calculated. Nociception to the acetone test was detected before (basal) and after treatments.

#### Hot plate test

Mice were placed on a hot plate (Ugo Basile) set at 50 ± 0.1 °C. The latency to the first hind paw licking/withdrawal was taken as an index of the nociceptive threshold and detected before (basal) and after treatments. Cutoff time was set at 30 s.

### Cell culture and isolation of primary sensory neurons

Human embryonic kidney (HEK293) cells stably transfected with the cDNA for human TRPA1 (hTRPA1-HEK293) or with the cDNA for human TRPV4 (hTRPV4-HEK293) and naive untransfected HEK293 cells (#RL-1573, American Type Culture Collection, ATCC) were cultured as previously described [24]. For mTRPA1 and mTRPV4, HEK293 cells were transfected with expression plasmid for mTRPV4 GFP-tagged [Trpv4 (NM_022017) Mouse Tagged ORF Clone MG226813, OriGene] or mTRPA1 GFP-tagged [Trpa1 (NM_177781) Mouse Tagged ORF Clone MG227099, OriGene]. Briefly, HEK293 cells were plated the day before transfection at 50–90% of confluence, then incubated for 72 h with Opti-MEM (#31985062, Thermo Fisher), DNA (OriGene), and TransIT (Mirus). After 72 h, the cells were selected with G418 (Thermo Fisher) (0.5 mg/ml) for 2 weeks. HEK293 and hTRPA1-HEK293 but not hTRPV4-HEK293, mTRPV4-HEK293, and mTRPA1-HEK293 were further authenticated.

Primary dorsal root ganglion (DRG) neurons were isolated from rats or *Trpa1*^*+/+*^ and *Trpa1*^*−/−*^ mice. Briefly, lumbosacral (L5–S2) ganglia were enzymatically digested using 2 mg/ml of collagenase type 1A with 1 mg/ml of trypsin for rats or with 1 mg/ml of papain for mice in Hanks’ Balanced Salt Solution (HBSS, 35 min, 37 °C). Ganglia were then transferred to warmed Dulbecco’s modified Eagle’s medium (DMEM) containing 10% fetal bovine serum (FBS), 10% horse serum, 2 mM l-glutamine, 100 U/ml penicillin, and 100 mg/ml streptomycin and mechanically dissociated in single cells. Neurons were filtered, centrifuged (6 min, × 1.200 rpm) at room temperature (RT), and resuspended in DMEM with added 100 ng/ml mouse-nerve growth factor and 2.5 mM cytosine-b-d-arabino-furanoside free base. The resuspended cells were plated on glass coverslips coated with poly-l-lysine (8.3 μM) and laminin (5 μM) and cultured for 3–4 days (37 °C) before calcium imaging or whole-cell patch-clamp recordings.

### Cellular recordings

Intracellular calcium was measured as previously reported [24]. Cells were exposed to H_2_O_2_ (30 μM–10 mM) or its vehicle (0.9% NaCl), allyl isothiocyanate (AITC, 10 μM) and the TRPV4 agonist, 4α-phorbol 12,13-didecanoate (4α-PDD, 1 μM) or their vehicle (0.01% DMSO). HC-030031 (30 μM), HC-067047 (10 μM), or their vehicles (3% and 1.5% DMSO, respectively); dithiothreitol (DTT, 10 mM) and 2-mercaptoethanol (β-ME, 50 mM) or their vehicles (0.9% NaCl); and PBN (30 μM) or its vehicle (0.9% NaCl) were applied 10 min before the stimuli. Results were expressed as the percentage of increase of ratio_340/380_ (*R*_340/380_) over the baseline normalized to the maximum effect induced by ionomycin (5 μM) added at the end of each experiments. Whole-cell patch-clamp recordings were performed as reported [24]. Currents were evoked in the voltage-clamp mode at a holding potential of − 60 mV; signals were sampled at 1 kHz and low-pass filtered at 10 kHz. Cells were stimulated with thalidomide, pomalidomide, and lenalidomide (all, 100 μM) or their vehicle (1% DMSO), and AITC (100 μM), capsaicin (CPS, 1 μM), and 4α-PDD (100 μM) or their vehicle (0.1% DMSO). Peak currents were normalized to cell membrane capacitance and expressed as mean of the current density (pA/pF) in averaged results.

### H_2_O_2_ measurement

The H_2_O_2_ content was determined in the tissues by using the Amplex Red® assay (Invitrogen). Tissues [hind paw, sciatic nerve, and lumbar (L4–L6) spinal cord] were collected and placed into modified Krebs/HEPES buffer containing (in mM): 99.01 NaCl, 4.69 KCl, 2.50 CaCl_2_, 1.20 MgSO_4_, 1.03 KH_2_PO_4_, 25.0 NaHCO_3_, 20.0 Na-HEPES, and 5.6 glucose, pH 7.4, minced, and incubated with Amplex Red (100 μM) and horseradish peroxidase (HRP, 1 U/ml) (1 h, 37 °C) protected from light. Fluorescence excitation and emission were at 540 and 590 nm, respectively. H_2_O_2_ production was calculated using H_2_O_2_ standard and expressed as micromoles per liter per milligram of dry tissue.

### Immunofluorescence

Mice were anesthetized, transcardially perfused with PBS (phosphate buffer saline), followed by 4% paraformaldehyde, and tissues (hind paw and sciatic nerve) were collected, post-fixed for 24 h, and paraffin embedded. Antigen retrieval was performed in sodium citrate buffer (10 mM sodium citrate, 0.05% Tween 20, pH 6.0) (20 min, 98 °C). The slides were then incubated with the 4-HNE primary antibody (#ab48506, HNEJ-2, mouse monoclonal, 1:40, Abcam) diluted in fresh blocking solution (PBS, pH 7.4, 2.5% normal goat serum, NGS) 1 h at RT, followed by a fluorescent polyclonal secondary antibody Alexa Fluor 594 (1:600; Invitrogen) 2 h at RT. Hind paws were also stained with TRPA1 (#ab58844, rabbit polyclonal, 1:400, Abcam), PGP9.5 (#ab8189, mouse monoclonal [13C4/I3C4], 1:600, Abcam), and S100 (#ab14849, mouse monoclonal [4B3], 1:300, Abcam), followed by fluorescent polyclonal secondary antibodies Alexa Fluor 594 and 488 (1600, Invitrogen) 2 h at RT. Slides were then coverslipped with mounting medium with 4′,6-diamidino-2-phenylindole (DAPI, #ab228549, Abcam).

Lumbar (L4–L6) spinal cord collected from perfused mice was placed overnight at 4 °C in 10% formalin, transferred to 30% sucrose overnight, frozen, and cryosectioned at 40 μm. Free-floating sections were incubated in PBS containing 0.1% Triton X-100 (TBS) and 2.5% NGS 1 h at room temperature, then in primary antibodies: TRPA1 (#58844, rabbit polyclonal, 1:400, Abcam), TRPV4 (#ACC-034, rabbit polyclonal, 1:200, Alomone Labs), 4-HNE (#ab48506, HNEJ-2, mouse monoclonal, 1:200, Abcam), GFAP (#MAB3402X, mouse monoclonal, clone GA5, Alexa Fluor® 488, 1:500, Merck), and CGRP (#PA1-85250, goat polyclonal, 1:500, Thermo Scientific) overnight at 4 °C. Sections were then incubated with the fluorescent polyclonal secondary antibodies Alexa Fluor 488 and Alexa Fluor 594 (1600; Invitrogen), and coverslipped using mounting medium with DAPI (Abcam). The specificity of TRPA1 and TRPV4 staining was confirmed by testing both antibodies in the spinal cord (a site where central endings of primary sensory neurons terminate) of mice with channel genetic deletion. Data show the absence of TRPA1 and TRPV4 staining in the dorsal horn of the lumbar spinal cord of *Trpa1*^*−/−*^ and *Trpv4*^*−/−*^ mice, respectively (Additional file 1: Fig. S4).

Fluorescence images were obtained using an AxioImager 2 microscope (Carl Zeiss). The fluorescence intensity of 4-HNE staining was evaluated by the image processing module of ZEN Pro (Carl Zeiss).

### Statistical analysis

Data are presented as mean ± SEM. For behavioral experiments with repeated measures, a two-way mixed model was used to compare the control and treated groups of mice at each time point tested, using the Bonferroni correction for multiple time points. The one-way ANOVA followed by the Bonferroni correction was used for comparison between multiple groups. Agonist potency was expressed as half maximal effective concentration (EC_50_). The data of mechanical threshold were log transformed before analysis to meet the parametric assumptions. Statistical analyses were performed using Prism 8 GraphPad software (GraphPad Software Inc.). *P* < 0.05 was considered statistically significant.

## Supplementary Information

**Additional file 1: FigS1.** Genetic deletion or pharmacological blockade of TRPV1 does not affect mechanical and cold hypersensitivity evoked by thalidomide. **FigS2.** Pomalidomide and lenalidomide evoke mechanical and cold allodynia. **FigS3.** Peripheral and central H_2_O_2_ contributes to thalidomide-induced mechanical allodynia. **FigS4.** Representative images of TRPA1 and TRPV4 protein staining in the mouse lumbar (L4-L6) spinal cord slices from *Trpa1*^*+/+*^ and *Trpa1*^*-/-*^ or *Trpv4*^*+/+*^ and *Trpv4*^*-/-*^ mice.

## Data Availability

All data generated or analyzed during this study are included in this published article and its Additional file 1.
