# Supplementary material for: Oxidative stress mediates thalidomide-induced pain by targeting peripheral TRPA1 and central TRPV4
Source: BMC Biol. 2020 Dec 14;18:197. doi: 10.1186/s12915-020-00935-9 (PMC7737339; doi:10.1186/s12915-020-00935-9)
Supplement: Supplementary file 1 — Additional file 1: FigS1. Genetic deletion or pharmacological blockade of TRPV1 does not affect mechanical and cold hypersensitivity evoked by thalidomide. FigS2. Pomalidomide and lenalidomide evoke mechanical and cold allodynia. FigS3. Peripheral and central H2O2 contributes to thalidomide-induced mechanical allodynia. FigS4. Representative images of TRPA1 and TRPV4 protein staining in the mouse lumbar (L4-L6) spinal cord slices from Trpa1+/+ and Trpa1-/- or Trpv4+/+ and Trpv4-/- mice. [file 12915_2020_935_MOESM1_ESM.docx]

**Additional File 1: Figures S1-S4**

**
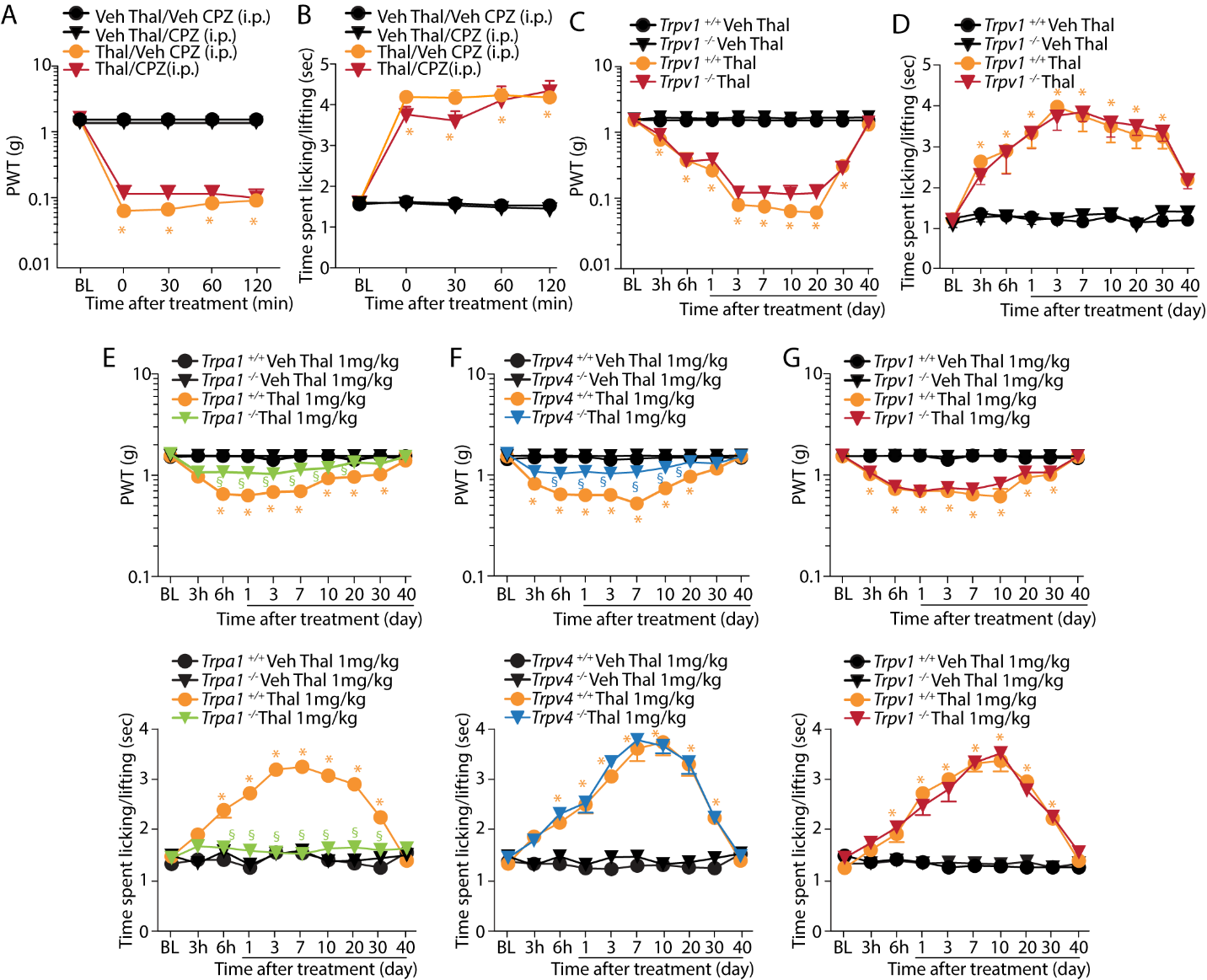
**

**Additional file 1: Fig. S1.** Genetic deletion or pharmacological blockade of TRPV1 does not affect mechanical and cold hypersensitivity evoked by thalidomide*.* **A** and **B**, Mechanical and cold allodynia at day 7 following intraperitoneal (i.p.) injection of thalidomide (Thal, 50 mg/kg) or Veh and after the administration of capsazepine (CPZ, 4 mg/kg, i.p.) or Veh. **C** and **D,** Time-dependent mechanical and cold allodynia following Thal (50 mg/kg, i.p.) or Veh in *Trpv1^+/+^* or *Trpv1^-/-^* mice. Time-dependent mechanical and cold allodynia following Thal (1 mg/kg, i.p.) in (**E**) *Trpa1^+/+^* or *Trpa1^-/-^*, (**F**) *Trpv4^+/+^* or *Trpv4^-/-^* and (**G**) *Trpv1^+/+^* or *Trpv1^-/-^* mice. BL, baseline. Data are mean ± SEM, n = 6 mice. **P*<0.05 *vs.* Veh Thal/Veh CPZ or *Trpv1^+/+^*/Veh Thal or *Trpa1^+/+^*/Veh Thal or *Trpv4^+/+^*/Veh Thal. ^§^*P*<0.05 *vs.* *Trpa1^+/+^*/Thal. Two-way ANOVA followed by Bonferroni’s post hoc test.

**
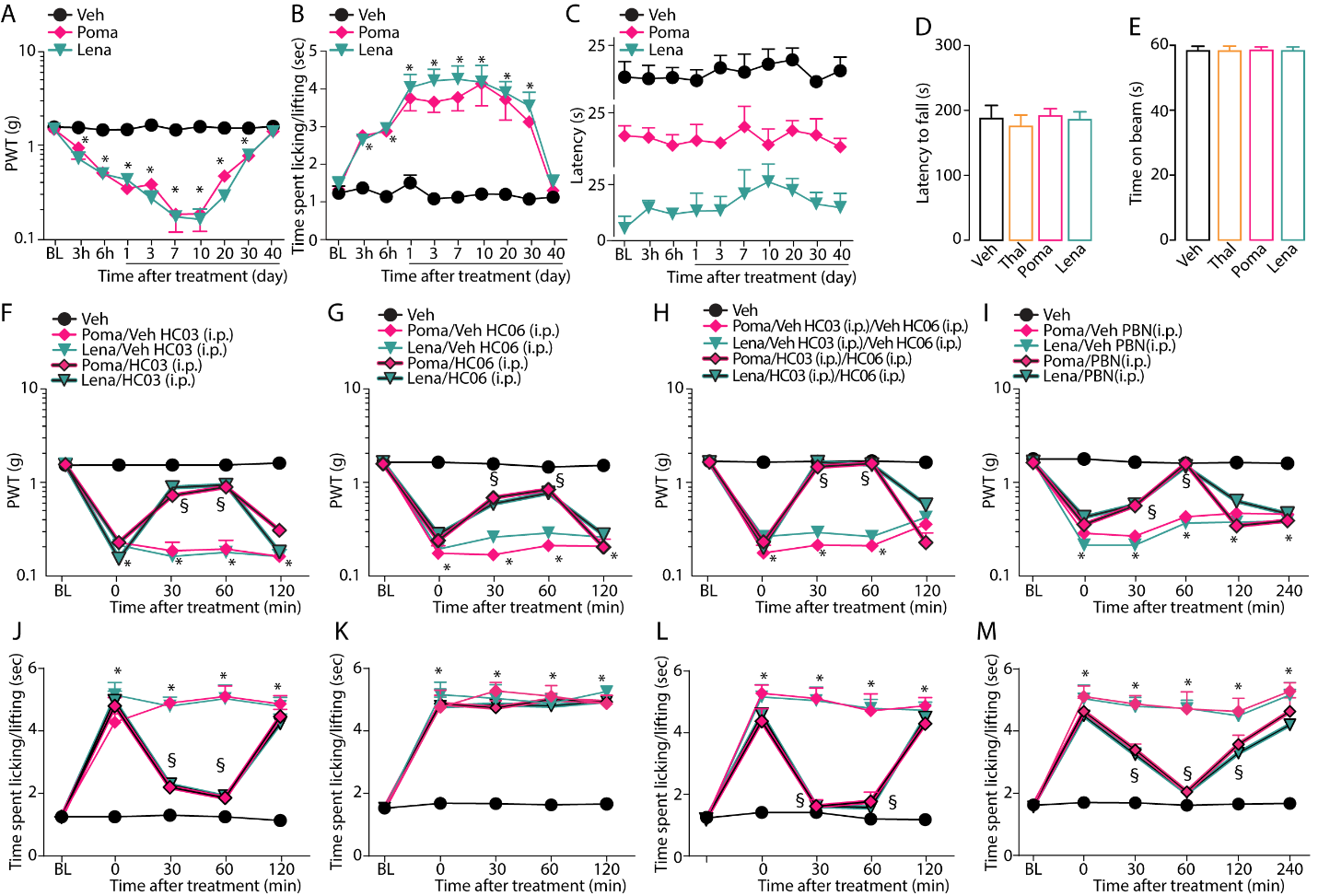
**

**Additional file 2: Fig. S2.** Pomalidomide and lenalidomide evoke mechanical and cold allodynia**. A**-**C,** Time-dependent mechanical, cold and heat hypersensitivity following intraperitoneal (i.p.) pomalidomide (Poma, 1 mg/kg), lenalidomide (Lena, 5 mg/kg) or Veh. **D**, Motor coordination on rotarod performance and **E**, balance beam test at day 7 following Thal (50 mg/kg, i.p.), Poma (1 mg/kg, i.p.), Lena (5 mg/kg, i.p.) or Veh. **F**-**H**, Mechanical allodynia at day 7 following Poma (1 mg/kg, i.p.) and Lena (5 mg/kg, i.p.) or Veh and after the administration of HC-030031 (HC03, 100 mg/kg, i.p.), HC-067047 (HC06, 10 mg/kg, i.p.) or a combination of HC03 (100 mg/kg, i.p.) and HC06 (10 mg/kg, i.p.) or Veh. **J-L**, Cold allodynia at day 7 following Poma (1 mg/kg, i.p.) and Lena (5 mg/kg, i.p.) or Veh and after the administration of HC03 (100 mg/kg, i.p.), HC06 (10 mg/kg, i.p.) or a combination of HC03 (100 mg/kg, i.p.) and HC06 (10 mg/kg, i.p.) or Veh. **I** and **M**, Mechanical and cold allodynia at day 7 following Poma (1 mg/kg, i.p.) and Lena (5 mg/kg, i.p.) or Veh and after the administration of phenyl-α-tert-butyl nitrone (PBN, 100 mg/kg, i.p.). Data are mean ± SEM, n = 6 mice. **P*<0.05 *vs.* Veh; ^§^*P*<0.05 *vs.* Poma/Lena/Veh HC03/Veh HC06 or Poma/Lena/Veh PBN. Two-way ANOVA followed by Bonferroni’s post hoc test.


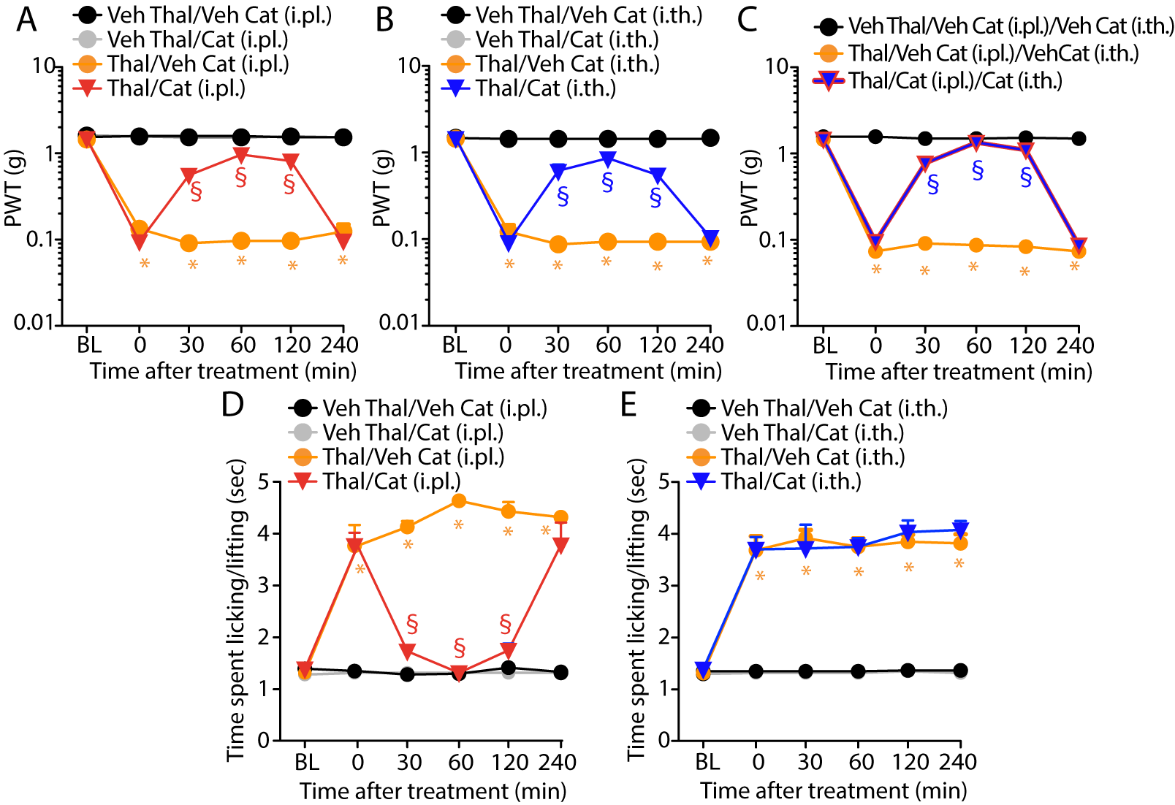


**Additional file 3: Fig. S3.** Peripheral and central H_2_O_2_ contributes to thalidomide-induced mechanical allodynia. **A-C,** Mechanical allodynia at day 7 following intraperitoneal (i.p.) thalidomide (Thal, 50 mg/kg) or Veh and after the administration of intraplantar (i.pl., 20 µl) or intrathecal (i.th., 5 µl) or a combination of i.pl. and i.th of catalase (Cat, 300 UI) or Veh. **D** and **E,** Cold allodynia at day 7 following Thal (50 mg/kg, i.p.) or Veh and after the administration of i.pl. or i.th. Cat (300 UI) or Veh. Data are mean ± SEM, n = 6 mice. **P*<0.05 *vs.* Veh Thal/Veh Cat; ^§^*P*<0.05 *vs.* Thal/Veh Cat; Two-way ANOVA followed by Bonferroni’s post hoc test.

**
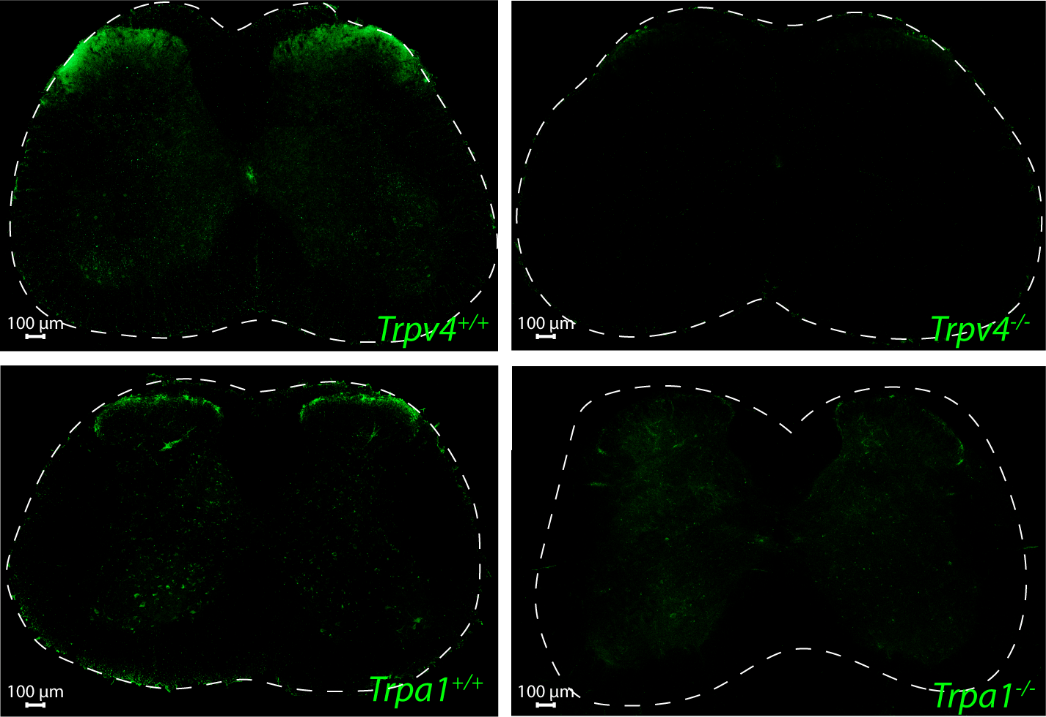
**

**Additional file 4: Fig. S4.** Representative images of TRPA1 and TRPV4 protein staining in the mouse lumbar (L4-L6) spinal cord slices from *Trpa1^+/+^* and *Trpa1^-/-^* or *Trpv4^+/+^* and *Trpv4^-/-^* mice.
